# Targeting Poly (ADP-Ribose) Polymerase Partially Contributes to Bufalin-Induced Cell Death in Multiple Myeloma Cells

**DOI:** 10.1371/journal.pone.0066130

**Published:** 2013-06-07

**Authors:** He Huang, Yang Cao, Wei Wei, Wei Liu, Shao-Yong Lu, Yu-Bao Chen, Yan Wang, Hua Yan, Ying-Li Wu

**Affiliations:** 1 Department of Hematology, Ruijin Hospital, Shanghai Jiao Tong University School of Medicine, Shanghai, China; 2 Department of Pathophysiology, Key Laboratory of Cell Differentiation and Apoptosis of the Chinese Ministry of Education, Shanghai Universities E-Institute for Chemical Biology, Shanghai Jiao Tong University School of Medicine, Shanghai, China; University of Pecs Medical School, Hungary

## Abstract

Despite recent pharmaceutical advancements in therapeutic drugs, multiple myeloma (MM) remains an incurable disease. Recently, ploy(ADP-ribose) polymerase 1 (PARP1) has been shown as a potentially promising target for MM therapy. A previous report suggested bufalin, a component of traditional Chinese medicine (“Chan Su”), might target PARP1. However, this hypothesis has not been verified. We here showed that bufalin could inhibit PARP1 activity *in vitro* and reduce DNA–damage-induced poly(ADP-ribosyl)ation in MM cells. Molecular docking analysis revealed that the active site of bufalin interaction is within the catalytic domain of PAPR1. Thus, PARP1 is a putative target of bufalin. Furthermore, we showed, for the first time that the proliferation of MM cell lines (NCI-H929, U266, RPMI8226 and MM.1S) and primary CD138^+^ MM cells could be inhibited by bufalin, mainly via apoptosis and G_2_-M phase cell cycle arrest. MM cell apoptosis was confirmed by apoptotic cell morphology, Annexin-V positive cells, and the caspase3 activation. We further evaluated the role of PARP1 in bufalin-induced apoptosis, discovering that PARP1 overexpression partially suppressed bufalin-induced cell death. Moreover, bufalin can act as chemosensitizer to enhance the cell growth-inhibitory effects of topotecan, camptothecin, etoposide and vorinostat in MM cells. Collectively, our data suggest that bufalin is a novel PARP1 inhibitor and a potentially promising therapeutic agent against MM alone or in combination with other drugs.

## Introduction

Poly(ADP-ribose) polymerase 1 (PARP1), a highly conserved DNA binding protein, is key in maintaining the genomic stability, repairing the DNA damage, and regulating transcriptional processes [1]–[2] by binding to cleaved DNA strands and catalyzing the NAD^+^-dependent addition of poly(ADP-ribose) (PAR) to target proteins. PARP1 influences other DNA repair enzymes to facilitate their function, too [3]. PARP1 inhibitor-mediated synthetic lethality was suggested to be important in breast and ovarian tumors with BRCA1 or BRCA2 gene mutations [4]. Arising from this research, PARP1-targeting therapy is gaining acceptance as an important strategy to treat tumor cells with BRCA1 or BRCA2 deficiencies. Several PARP1 inhibitors are presently in clinical trials, and PARP1 inhibitors are being recognized as useful chemosensitizers not only in patients with BRCA–deficiency tumors, but also for patients in which tumors have general homologous recombination defects [5].

The plasma cell malignancy, multiple myeloma (MM), is the second most common hematologic cancer in world. Despite advances in understanding the mechanism underlying MM and the development of novel therapeutic agents, such as bortezomib, lenalidomide and thalidomide, MM remains incurable [6]. Thus, further elucidation of MM pathogenesis and the identification of novel targets are urgently needed. Interestingly, Neri *et al.* recently reported that elevated PARP1 expression was correlated with poor survival in MM and bortezomib-induced “BRCAness” (tumor cells with the homologous recombination deficiency) showed synergistic anti-tumor effects when paired with PARP1 inhibitors for killing MM cells [7]. Therefore, PARP1 is a potentially promising target for MM therapy.

Bufalin is a cardiotonic steroid isolated from the skin and parotid venom glands of toads of the Bufo species (*Bufo bufo gargarizans*). Bufalin is commonly used in the practice of Chinese medicine, comprising the active ingredient in Chan Su and a major component of Liu-Shen-Wan [8] and Kyushin [9]. Bufalin has cardiotonic, anaesthetic, hypertensive, respiration-inducing, and antineoplastic activities. The antitumor activity of bufalin has been demonstrated in various cancer cells, including leukemia [10]–[13], and cancers of the prostate [14], ovaries, endometrium [15], gastrointestinal system [16], pancreas, lung [17], and liver cells [18], [19]. These antitumor effects have been mainly attributed to the induction of apoptosis and cell cycle arrest. Accumulating evidence on bufalin revealed that it stimulates reactive oxygen species and inhibits of NF-κB, STAT3, and AKT signaling pathways, all of which are contributed to its antitumor effects. The Na^+^-K^+^-ATPase has been shown to be a direct target of bufalin [20], [21], which inhibits its activity, but recent studies suggest that bufalin can induce cellular signaling events independent of Na^+^-K^+^-ATPase suppression [22]–[24].

Here, we show that PARP1 is a novel target of bufalin, and that PARP1 partially contributes to bufalin-induced cell death in MM cells. Moreover, bufalin significantly enhances MM cell sensitivity to several chemotherapeutic drugs. Thus, bufalin holds promise for the treatment of MM alone or perhaps combined with other agents.

## Results

### Bufalin interacts with PARP1 and inhibits its activity *in vitro*

**Figure 1:**
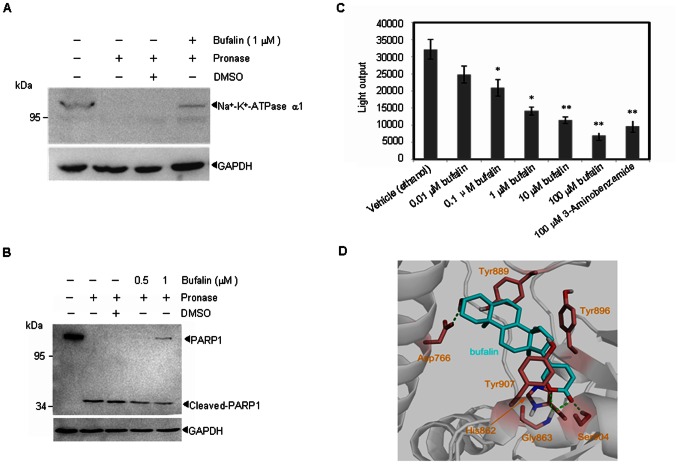
Bufalin interacts with PARP1 and inhibits PARP1 activity *in vitro*. (A–B) Whole cell lysates of U266 cells were incubated with bufalin followed by digestion with pronase according to “Materials and Methods”. Then, the degree of Na^+^-K^+^-ATPase (A) or PARP1 (B) degradation was determined by western blot. All experiments were repeated for three times. (C) PARP1 activity was measured as described in the “Materials and Methods” in the presence or absence of bufalin (concentrations indicated), 3-Aminobenzamide (100 µM) was used as a positive control. All values represent means ± S.D. of three independent experiments, each performed in triplicate (^*^ *P*<0.05, ^**^ *P*<0.01, compared with the vehicle control). (D) The predicted binding mode of bufalin to human PARP1. PARP1 and bufalin are respectively displayed as cartoon and stick. Side chains of crucial residues in the binding site are shown as stick and labeled. Hydrogen bonds between bufalin and PARP1 are depicted with dotted green lines.

Recently, PARP1 has been shown to be a potential target for MM therapy, and PARP1 has been suggested to interact with bufalin [25]. Using Drug Affinity Responsive Target Stability (DARTS), we investigated the possible interaction between bufalin and PARP1. To demonstrate the validity of the technique used to measure the bufalin-PARP1 interaction, we studied the established interaction between bufalin and the Na^+^-K^+^-ATPase (Fig. 1A), rapamycin and FKBP12 [26], [27] (Fig. S1A). As shown in Fig. 1B and Fig. S1B, 1 µM bufalin significantly protected PARP1 protein from digestion by pronase, indicating that bufalin interacts directly with PARP1 *in vitro*.

Next, we examined the effects of bufalin on PARP1 activity *in vitro*. Highly purified PARP1 protein was pre-incubated with various concentrations of bufalin or the PARP1 inhibitor 3-Aminobenzamide. Then, PARP1 activity was measured using a PARP1 assay kit. As shown in Fig. 1C, bufalin suppressed PARP1 enzymatic activity even at 10 nM, with a dose-dependent increase in inhibition. These data suggest that bufalin inhibits PARP1 activity *in vitro*.

To investigate how bufalin interacts with or potentially binds to PARP1, we carried out docking simulations. The ligand bufalin was encapsulated by Asp766, His862, Gly863, Tyr889, Tyr896, Ser904, and Tyr907 residues. The side chain carboxyl group of Asp766 forms a hydrogen bond with the terminal hydroxyl group of the ligand. The backbone amide group of Gly863 donates bifurcated hydrogen bonds to the two oxygen atoms of α-pyrone moiety of the ligand, while the endocyclic oxygen atom of the α-pyrone moiety creats of a hydrogen bond with the side chain hydroxyl group of Ser904. In addition, His862 and Tyr907 are engaged in π-π stacking interactions with the α-pyrone moiety, respectively (Fig. 1D). Of note, by amino acid sequence processing, 6 tryptic peptiedes (His862, Gly863, Tyr889, Tyr896, Ser904, and Tyr907) are matched across the catalytic domain protein sequences of PARP1 (amino acids 788 to 1014), the interaction region lies in the catalytic domain of PAPR1, supporting the inhibition activity of bufalin on PARP1.

### Bufalin inhibits PARP1 activation in cells

**Figure 2:**
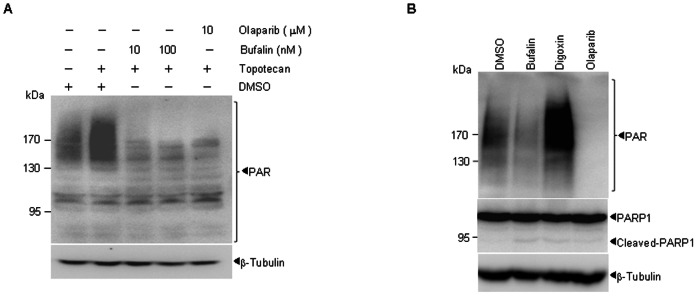
Bufalin functions as an inhibitor of PARP1 in cells. (A) H929 cells were pretreated with indicated concentrations of bufalin or olaparib for 1 h, and then exposed to 100 nM topotecan for another 1 h. Poly (ADP-ribosyl)ation was measured by western blot. (B) H929 cells were cultured with DMSO, 20 nM bufalin, 100 nM digoxin and 20 µM olaparib respectively, and then endogenous PAR expression was quantified by western blot at 48 h. All experiments were repeated for at least three times.

Based on the above observation, we assumed that bufalin might inhibit PARP1 activity in cells. To this end, H929 cells were pretreated with DMSO, bufalin or olaparib, and then treated with topotecan (a topoisoerase enzyme I inhibitor) [28] which can activate PARP1. If bufalin can inhibit the activity of PARP1, it should inhibit the DNA damage repair-induced increment of PAR [29]–[31]. Indeed, similar to findings with the PARP1 inhibitor olaparib, pretreatment of bufalin inhibited topotecan-induced accumulation of PAR (Fig. 2A). These data indicated that bufalin might inhibit PARP1 activity in cells. It is well known that bufalin is a Na^+^-K^+^-ATPase inhibitor, so to investigate the relationship between Na^+^-K^+^-ATPase inhibition and PARP1 inhibition, H929 cells were treated for 48 h with bufalin, digoxin, or olaparib, respectively. Interestingly, when compared with bufalin and olaparib [32], digoxin, a known Na^+^-K^+^-ATPase inhibitor, markedly increased PAR expression in H929 cells (Fig. 2B), suggesting that bufalin-induced PAR reduction may not due to the inhibition of Na^+^-K^+^-ATPase.

### Bufalin markedly inhibits MM cells proliferation

**Figure 3:**
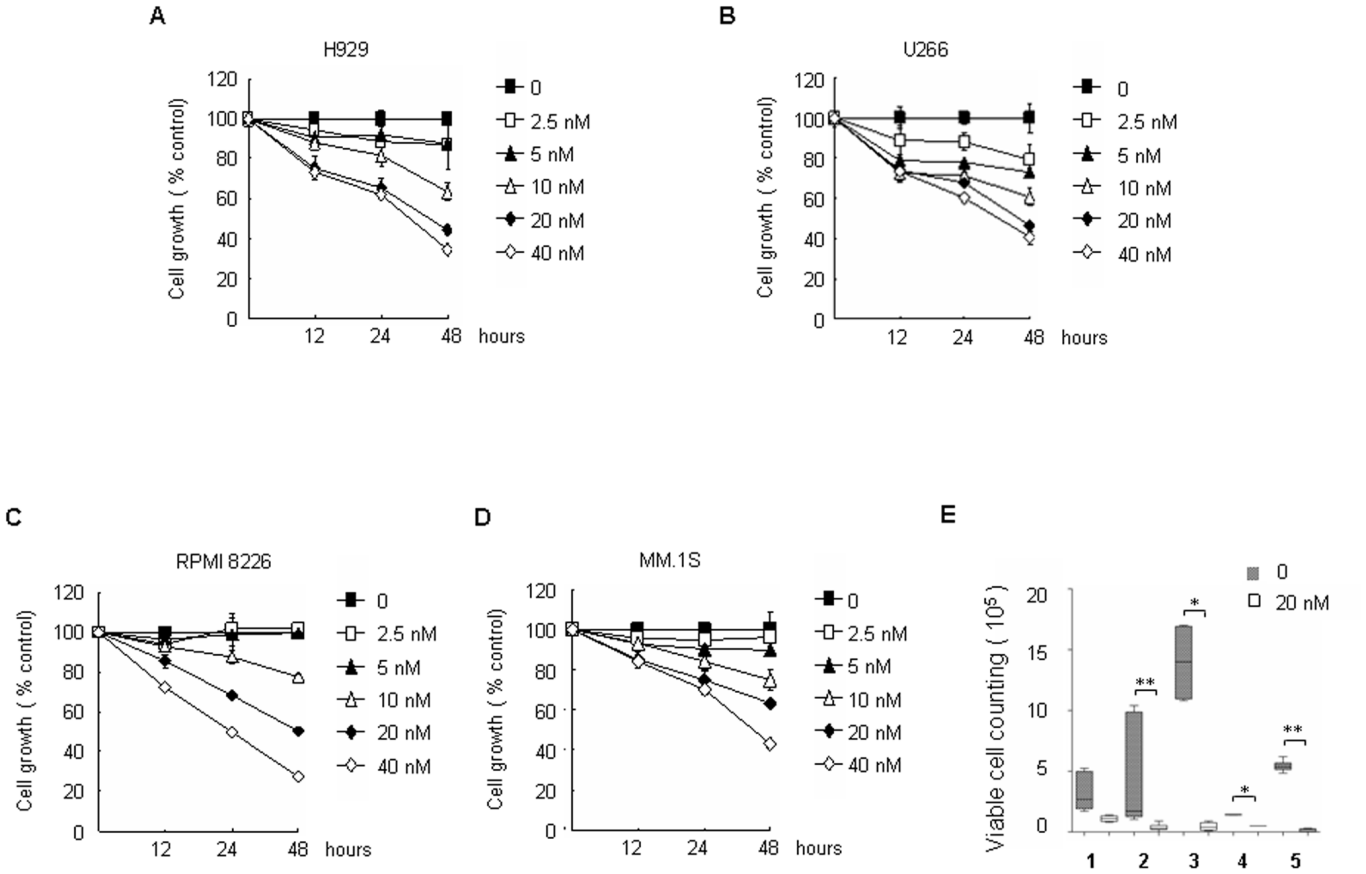
Bufalin inhibits MM cells proliferation. (A–D) Cells were seeded in 96-well plates and treated with different concentrations of bufalin for 12, 24 and 48 h. Bufalin-induced effects on cell proliferation were measured by CCK8 assay. All values represent means ± S.D. of three independent experiments, each performed in triplicate. (E) CD138^+^ cells isolated from MM patients were incubated with 20 nM bufalin for 15 days as described in “Materials and Methods”. Cells were collected, and viable cells were counted under a microscope using 0.4% Trypan blue stain. A box plot showing the 5th and 95th percentiles, together with the median, with showing the minimum and maximum difference in the percent slide-positivity rates among duplicates, the Mann-Whitney-Wilcoxon test was used to compare untreated control and treated samples (^*^ *P*<0.05, ^**^ *P*<0.01, compared to the controls).

To examine MM cells sensitivity to bufalin, MM cell lines (NCI-H929, U266, RPMI8226 and MM.1S) were treated with various concentrations of bufalin for 12, 24 and 48 h, and cytotoxicity was assessed by CCK8 assay, respectively. Bufalin inhibited the MM cell growth in a time- and dose-dependent manner (IC_50_ ∼20 nM at 48 h). MM.1S cells were less sensitive to bufalin than H929, U266, and RPMI8226 cells (Fig. 3A–D). To further determine the effect of bufalin on primary MM cells, the CD138^+^ cells from 5 MM patients were isolated and cultured in methylcellulose medium in the presence or absence of bufalin. As shown in Fig. 3E, although different sensitivities were observed across samples, bufalin treatment significantly reduced the survival of MM cells, including ones derived from the relapsed MM cases.

### Bufalin induces apoptosis and cell cycle arrest in MM cells

**Figure 4:**
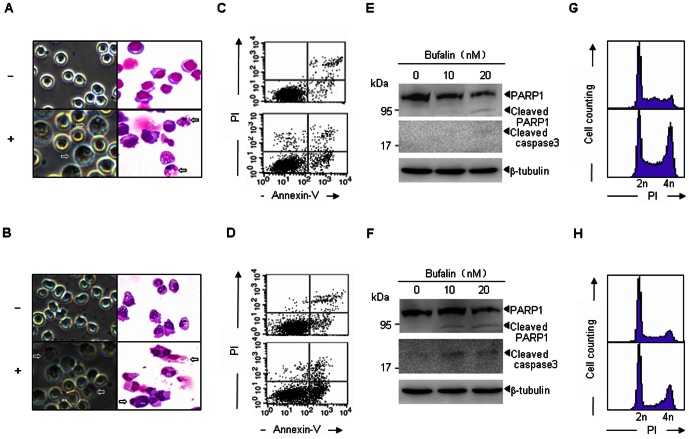
Bufalin induces apoptosis and cell cycle arrest in MM cells. H929 (A, C, E, G) and U266 (B, D, F, H) cells were treated with or without 20 nM bufalin for 48 h, and cell morphology (A–B) was monitored by phase contrast microscope (40×, left) or Wright's staining (100×, right). Arrowheads indicated apoptotic cells. Annexin-V-positive cells (C–D) were quantified by flow cytometry analysis. Indicated proteins were analyzed by western blot analysis (E–F). Cell cycle distribution was determined by flow cytometry analysis of DNA content (G–H). Each experiment was performed in triplicate and repeated at least three times.

Establishing that bufalin can inhibit the MM cell proliferation and decrease viability, we next examined the apoptotic effects of bufalin in H929 and U266 cells. Cells were treated with 20 nM bufalin for 48 h, and morphologic features of apoptotic cells, including nuclear shrinkage and apoptotic body formation were observed in the two tested cell lines (Figs. 4A and 4B). The percentage of apoptotic cells were further evaluated by Annexin-V/PI double staining. As shown in Fig. 4C and 4D, bufalin treatment induced ∼33% and 76% Annexin-V positive cells in H929 and U266, respectively. Supporting these findings, cleavage of caspase3 and PARP1 were also observed in bufalin-treated cells (Figs. 4E and 4F). We also investigated the effect of bufalin on cell cycle distribution, and found that G_2_-M arrest was observed in H929 and U266 cells, most profoundly in H929 cells (Fig. 4G and 4H). Thus, bufalin can induce apoptosis and G_2_-M arrest in H929 and U266 cells.

### Overexpression of PARP1 partially inhibits bufalin-induced apoptosis in MM cells

**Figure 5:**
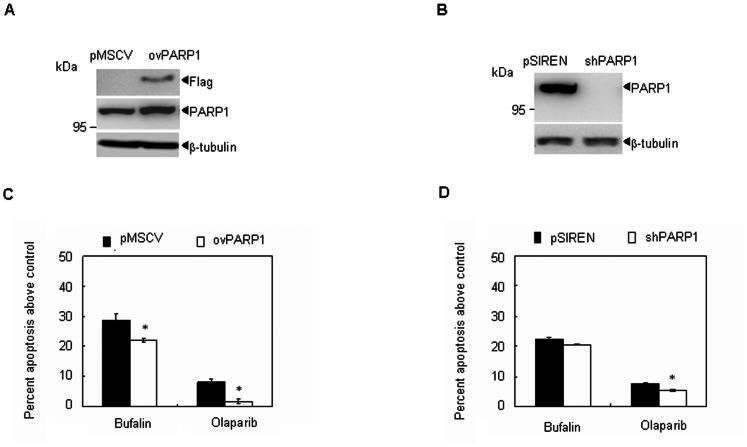
The role of PARP1 in bufalin-mediated apoptosis in MM cells. (A–B) H929 cells were stablely transfected with the control empty vector (pMSCV) or a PARP ovexpression plasmid (ovPARP1), or with the control nonspecific shRNA (pSIREN) or the shRNA against PARP1 (shPARP1), PARP1 expression was measured by western blot. (C) Indicated cells were treated with 20 nM bufalin or 20 µM olaparib for 48 hours. Annexin-V-positive cells were counted using flow cytometry. All values represent means ± S.D. of three independent experiments, each performed in triplicate (^*^ *P*<0.05, ^**^ *P*<0.01, compared to vector transfected controls).

To investigate the possible role of PARP1 inhibition in bufalin-induced apoptosis, PARP1 was overexpressed (Fig. 5A) in H929 cells. Upon treatment with bufalin, H929 cells with PARP1 overexpression had significantly decreased Annexin-V-positive cells than vector-transfected H929 cells (Fig. 5C). However, PARP1 knockdown did not enhance bufalin-induced apoptosis, similar to what was observed in olaparib-treated cells (Fig. 5B and 5D). These data indicate that PARP1 inhibition partially contributes to bufalin- induced apoptosis.

### Bufalin enhances chemosensitivity of H929 cells

**Figure 6:**
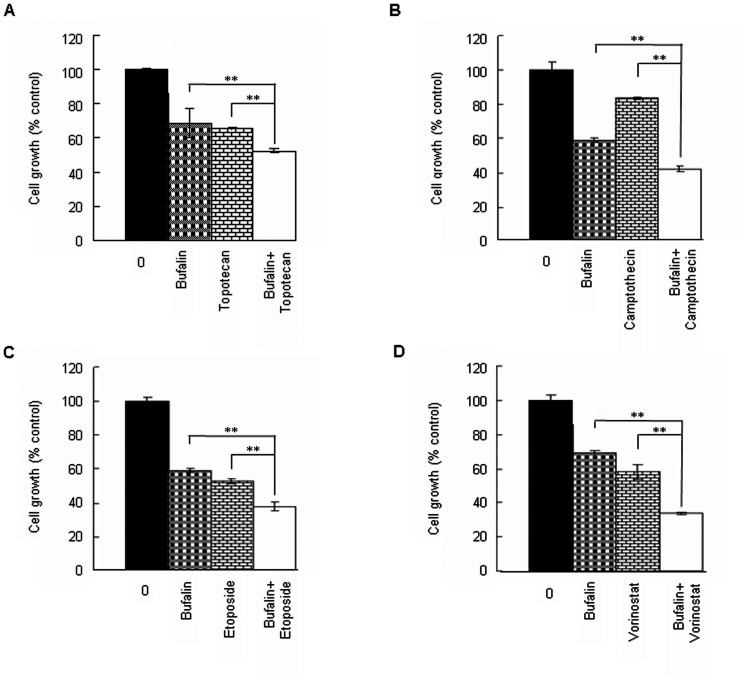
Bufalin enhances chemosensitivity of H929 cells. (A–D) H929 cells were treated with 10 nM bufalin alone, or in combination with 50 nM topotecan, 10 nM camptothecin, 2.5 µM etoposide, and 0.5 µM vorinostat, respectively for 48 h. Cell proliferation was measured by CCK8 assay. All values represent means ± S.D. of three independent experiments, each performed in triplicate (.^**^ *P*<0.01, compared to single drug treatment).

PARP1 inhibitors have been shown to improve the therapeutic efficacy of several commonly used chemotherapeutics. Because bufalin can inhibit the PARP1 activity *in vitro* and in cells, we next studied its potential to be used as chemosensitizer. H929 cells were treated with bufalin alone or in combination with topotecan, camptothecin, etoposide and the HDAC inhibitor vorinostat [33]–[35] for 48 h, respectively. As shown (Figs. 6A–D), bufalin significantly potentiated the cell proliferation suppression induced by the chemotherapeutics.

## Discussion

In this study, we demonstrate that bufalin can directly bind to and inhibit PARP1 activity, and that PARP1 inhibition contributes partially to bufalin-induced apoptosis in MM cells. Furthermore, we showed that bufalin could be used as a chemotherapeutic sensitizer to enhance the efficacy of some drugs.

MM is an incurable plasma cell malignancy, which has prompted efforts toward developing novel therapeutics to improve patient outcomes. Bufalin has been shown to be effective in multiple cancer cell lines. However, the potential effect of bufalin on MM has not been verified. Because PARP1 represents an attractive target for MM therapy and Ma *et al.* predicted that PARP1 was a potential target of bufalin via the molecular docking method [25], we investigated whether bufalin can target PARP1 and exert its anti-myeloma activity. We showed that bufalin can target PARP1, which is supported by following evidence: (a) bufalin can protect PARP1 from protease-induced degradation, indicating that bufalin may bind to PARP1. DARTS is an established and valid methodology for identifying and studying protein-ligand interactions. DARTS is comparatively simple and can be performed using crude lysates with native, unmodified small molecules. The reliability of this method was established by documenting the protective effect of bufalin on the Na^+^-K^+^-ATPase and rapamycin on FKBP12 protein; (b) the *in vitro* assay showed that bufalin could significantly inhibit the PARP1 activity in a dose-dependent manner; (c) consistent with its inhibitory activity *in vitro*, bufalin significantly inhibited PAR accumulation in MM induced by topotecan, a drug that damages DNA. Similar results were observed in olaparib-treated cells. This effect was not due to the inhibition of Na^+^-K^+^-ATPase, when digoxin alone can increase the PAR accumulation; (d) a previous report indicated that DNA repair was delayed in bufalin-pretreated cells after X-rays irradiation [36]. We found that bufalin induced G_2_-M arrest in MM cells [37], an observation which may also reflect the inhibition of PARP1 activity; (e) PARP1 consists of three distinct domains: the DNA-binding domain, which plays a key role in recognition of DNA lesions; the auto-modification domain, which provides protein-gathering sites; and the catalytic domain, which poly(ADP-ribosyl)ates histones and PARP1 itself [38]. The molecular docking study suggested that bufalin preferentially interacts with the catalytic domain of PARP1. Although further mutation or co-crystal studies are needed to elucidate the interaction between bufalin and PARP1, these data support that PARP1 can be inhibited by bufalin. Collectively, our data indicate that bufalin binds to PARP1 and inhibits its activity *in vitro* and in cells.

Consistent with the reported wide anti-tumor activity, bufalin can also induce apoptosis in H929 and U266 cells, as evidenced by the apoptotic body formation, an increase in Annexin-V-positive cells, and caspase3 activation. Importantly, bufalin can effectively decreases the proliferation of primary myeloma cells, including cells from a relapsed patient. As PARP1 inhibition has been associated with apoptosis and necrosis, therefore, bufalin may target PARP1 for its anti-MM effect. However, overexpression of PARP1 only partially inhibits bufalin-induced apoptosis and PARP1 knocking-down has little influence on bufalin-induced apoptosis. This may be explained by the fact that bufalin also targets the Na^+^-K^+^-ATPase, which plays a dominant role in bufalin-induced cell death. Because of the crucial role of PARP1 in response to DNA damage, PARP1 inhibition was identified as a potential therapeutic target to enhance the efficacy of DNA damage agents, or to certain cancer cells lacking homologous recombination repair. Therefore, we tested the combinations effect of bufalin with several DNA damaging agents including topotecan, camptothecin, etoposide and HDAC inhibitor vorinostat. We found that bufalin significantly enhanced the cytotoxicity of these drugs, revealing chemosensitizing activity.

In conclusion, we demonstrate for the first time that PARP1 is a novel target of bufalin and that bufalin is effective in inducing cell death in MM cells. Considering the fact that bufalin containing “Huachansu”, an injectable form of “Chan Su”, had low toxicity and few side effects in patients who suffered hepatocelluar and pancreatic cancer [17], we propose that bufalin to be a promising candidate for MM therapy in clinical applications. Furthermore, as a PARP1 inhibitor, bufalin might be effective in those cancer cells with BRCA1/2 deficiencies, a concept which warrants further investigation.

## Materials and Methods

### Cell culture

The human multiple myeloma cell lines NCI-H929, U266, RPMI8226, MM.1S (purchased from ATCC) were cultured in RPMI 1640 medium (Sigma-Aldrich, St. Louis, MO), supplemented with 10% (vol/vol) heat-inactivated fetal bovine serum (FBS; hyClone, Logan, UT), penicillin (100 IU/mL) and streptomycin (100 µg/mL) in a humidified incubator at 37°C and 5% CO_2_/95% air.

### Small molecular compounds

Bufalin ((3beta, 5beta)-3, 14-Dihydroxy-bufa-20, 22-dienolide) was purchased from Tauto Biotech Co., Ltd., Shanghai China, with >97.6% purity as judged by HPLC analysis. Rapamycin, olaparib, camptothecin, etoposide, and vorinostat were purchased from Selleck Chemicals, while topotecan and digoxin were purchased from Sigma-Aldrich. All above drugs were dissolved in dimethyl sulfoxide (DMSO) to make stable stock solution and stored at −20°C.

### Small-molecule target identification

Drug affinity responsive target stability (DARTS), a general methodology for identifying and studying protein-ligand interaction was employed as a vitro method to define the PARP1-bufalin interaction [39]. MM cell pellets were lysed in standard Triton X-100 lysis buffer (50 mM Tris-HCl pH 7.5, 200 mM NaCl, 0.5% Triton X-100, 10% glycerol, 1 mM DTT), supplemented with protease inhibitor cocktails (Sigma). The cell lysates incubated with drugs or vehicle in room temperature for 50 minutes, and the above mixture continued to be digested by pronase (10 mg/ml stock solutions in water - aliquots stored at −20°C) at appropriate ratio dissolved in TNC buffer (50 mM Tris-HCl pH 8.0, 50 mM NaCl, 10 mM CaCl_2_) for 30 minute. The reaction was stopped by the addition of concentrated SDS-PAGE loading buffer to final 1×, mixed well and boiled immediately. Samples were subjected to Western blot.

### PARP enzyme activity assay in vitro

The *in vitro* inhibitory effect of bufalin on PARP1 enzymatic activity was determined using HT Universal Chemiluminescent PARP Assay Kit (Trevigen, Inc.). This kit measures the incorporation of biotinylated PAR onto histone proteins in a strip well format. According to manufacture's instruction, different concentrations of bufalin was incubated with the purified PARP enzyme (PARP-HSA) (0.5 u/well) for 10 min, then the mixture were incubated with PARP cocktail containing biotinylated NAD, and activated DNA in histone-coated stripe wells for 60 min. After washing, Strep-HRP was added and incubated for 60 min, followed by adding the mixture of PeroxyGlow™ A and B. The chemiluminescent signal was examined on the Synergy H4 Hybrid Microplate Reader plate reader. To avoid the vehicle (DMSO) influence [40], we dissolved bufalin in ethanol instead of DMSO. 3-Aminobenzamide (PARP1 inhibitor) was used as a positive control.

### Molecular docking

Molecular docking of the ligand bufalin to the human PRAP1 active site was carried out using the AutoDock4.2 software. The coordinates of human PARP1 were obtained from the refined X-ray crystal structure of 3L3M.pdb [41], which is available from the Protein Data Bank. Tools (ADT) for PARP1. Kollamn united partial atomic charges were then assigned for the protein, and the AutoDock atom types were defined using ADT. For the ligand bufalin, all hydrogen atoms were added, and the default root, rotatable bonds, and torsion of bufalin were set through TORSDOF module in ADT. The grid center was defined at the centroid of the inhibitor A96, and the number of grid points in the x, y, and z directions were set to 60, 60, and 60 with a spacing value 0f 0.375 Å using AutoGrid. The distance-dependent function of the dielectric constant was employed to calculate the energetic maps. The Lamarckian genetic algorithm was used for ligand conformational search. We used the same docking parameters as previously reported [42]. Finally, 100 independent docking runs were conducted. The docked conformations were ranked into clusters based on the binding energy. The results were clustered using a tolerance of 1.0 Å root-mean-square deviations. The structure with the largest number of neighbors within this threshold was considered the first, largest cluster. The lowest-energy complex in the cluster with the largest number of neighbors was selected for analysis.

### Western blot

MM cells were harvested, washed with PBS and lysed with lysis buffer (62.5 mM Tris-HCl, pH 6.8, 100 mM DTT, 2% SDS, 10% glycerol). Cell lysates were centrifuged at 20,000 g for 10 minutes at 4°C, and proteins in the supernatants were quantified. Protein extracts were equally loaded to 6% to 15% SDS–polyacrylamide gels, electrophoresed, and transferred to nitrocellulose membrane (Amersham Bioscience, Buckinghamshire, United Kingdom). After blocking with 5% nonfat milk in PBS for 2 h at room temperature, the membranes were incubated with antibodies against Na^+^-K^+^-ATPase α1 (Bioworld Technology, Inc.), cleaved caspase 3 (Cell Signaling, Beverly, MA), PARP1 (Santa Cruz Biotechnology, CA) and PAR (Trevigen, Inc.) overnight at 4°C, followed by HRP-linked secondary antibody for 1 h at room temperature. The signals were detected by chemiluminescence phototope-HRP kit (Millipore) according to manufacturer's instructions, and β-tubulin (Sigma) was probed as an internal control.

### Cell proliferation and cell viability assay

MM cells (0.1–3×10^5^ per 200 µl) were seeded into 96-well plates and incubated with various drug concentrations in triplicates for 48 h. Cell counting kit-8 (CCK8) (Dojindo, Kumamoto, Japan) was used to measure cell proliferation as described previously [43]. Each experiment was conducted in triplicate and repeated three times. CD138^+^ cells from MM patients were mixed well with 0 nM or 20 nM bufalin, and then the mixture including 2×10^5^ cells were plated in semisolid methylcellulose progenitor culture (Methocult H4434; Stem Cell Technologies, Vancouver, BC, Canada). After 15 days incubation at 37°C in a fully humidified atmosphere of 5% CO_2_, the culture media was washed twice with PBS, and the viable cells were counted by 0.4% trypan blue dye exclusion assay.

### Patient samples

Patient samples were obtained with informed consent the under Clinical Investigational Reviewing Board of Shanghai Second Medical University, Shanghai, China which approved the procurement protocol. Mononuclear cells were isolated on a Ficoll-hypaque (Pharmacia, Piscataway, NJ) density gradient using standard procedures which separated bone marrow from MM patients. CD138^+^ cells were further isolated by positive selection utilizing EasySep CD138^+^ magnetic nanoparticles per the manufacturer's instructions (StemCell Technologies, Vancouver, BC). Cell viability determined by trypan blue exclusion was greater than 90%.

### Cell morphology analysis

Cells were observed and photographed directly in flasks by phase contrast microscope, or were collected onto slides by cytospin (Shandon, Runcorn, United Kingdom), stained with Wright staining, and examined under a light microscope.

### Cell apoptosis assays

Cell apoptosis was measured with the Annexin-V Apoptosis detection kit (BD Pharmingen) following the manufacturer's instructions. Annexin-V-positive and propidium iodide-negative cells were considered to be in the early apoptotic phase and those having positive staining both for Annexin-V and propidium iodide were consider to be in stages of late apoptosis or necrosis. All data were collected, stored, and analyzed by LYSIS II software (BD Biosciences).

### Cell cycle analysis

Bufalin-treated cells were harvested and fixed with 75% cold ethanol at −20°C over 24 h. Then cells were incubated with RNase (100 mg/ml) and stained with propidium iodide (PI; Sigma) (250 µg/ml). Fluorescent intensities of stained DNA were quantified by flow cytometry (Becton Dickinson).

### RNA interference

The retrovirus vector for PARP1 protein suppression by short hairpin RNA (shRNA) interference was generated as described previously [44]. Briefly, a retrovirus with target sites in the PARP1 gene (5′-AAGATAGAGCGTGAAGGCGAA-3′) and non-target control shRNA (NC)-containing plasmids were packaged in HEK293T cells by co-transfecting them with pSIREN-RetroQ, pEQPAM (containing gag-pol, produced by Dr. Lishan Su at UNC, Chapel Hill, NC) and VSVG (Clontech, T-334350). After transfection for 48 h, the viral supernatant was collected, filter-sterilized and added to H929 cells (2×10^5^ cells/well) in 6-well plates in a medium containing 8 µg/ml of polybrene (Millipore, TR-1003-G), Puromycin (2.0 µg/ml, Calbiochem, 540411) was used to select the stably transfected cells at 96 h.

### Plasmids

Full-length cDNA for human PARP1 was a generous gift from Han's Lab at Xiamen University, China. The full-length PARP1 cDNA was sub-cloned into the pMSCV vector by PCR amplification using specific primers (PARP1-Bgi (F): 5′-AGCTAGATCTATGGCGGAGTCTTCGGATAAG-3′; PARP1-Xho (R): 5′-AGCTCTCGAGTTACCACAGGGAGGTCTTAAAAT-3′), and a flag tag was added at the N-terminal. The integrity of the amplified cDNA fragment in pMSCV-flag-PARP1 vector was verified by DNA sequencing.

### Statistical analysis

Data from patient samples was analyzed with the Mann-Whitney-Wilcoxon test (SAS software, version 8.0). Data from cell lines was analyzed with the Student T-test. *P* value <0.05 was considered to be statistically significant.

## Supporting Information

Figure S1 **Using DARTS method to demonstrate the interaction between FKBP12 and rapamycin, or PARP1 and bufalin.** H929 cell lysates were incubated with rapamycin (A) or bufalin (B) followed by digestion with pronase according to “Materials and Methods”. Then, the degree of FKBP12 and PARP1 protein degradation was determined by western blot. All experiments were repeated for three times.
(TIF)
