# Supplementary figures and images for: Targeting Poly (ADP-Ribose) Polymerase Partially Contributes to Bufalin-Induced Cell Death in Multiple Myeloma Cells
Source: PLoS One. 2013 Jun 7;8(6):e66130. doi: 10.1371/journal.pone.0066130 (PMC3676346; doi:10.1371/journal.pone.0066130)

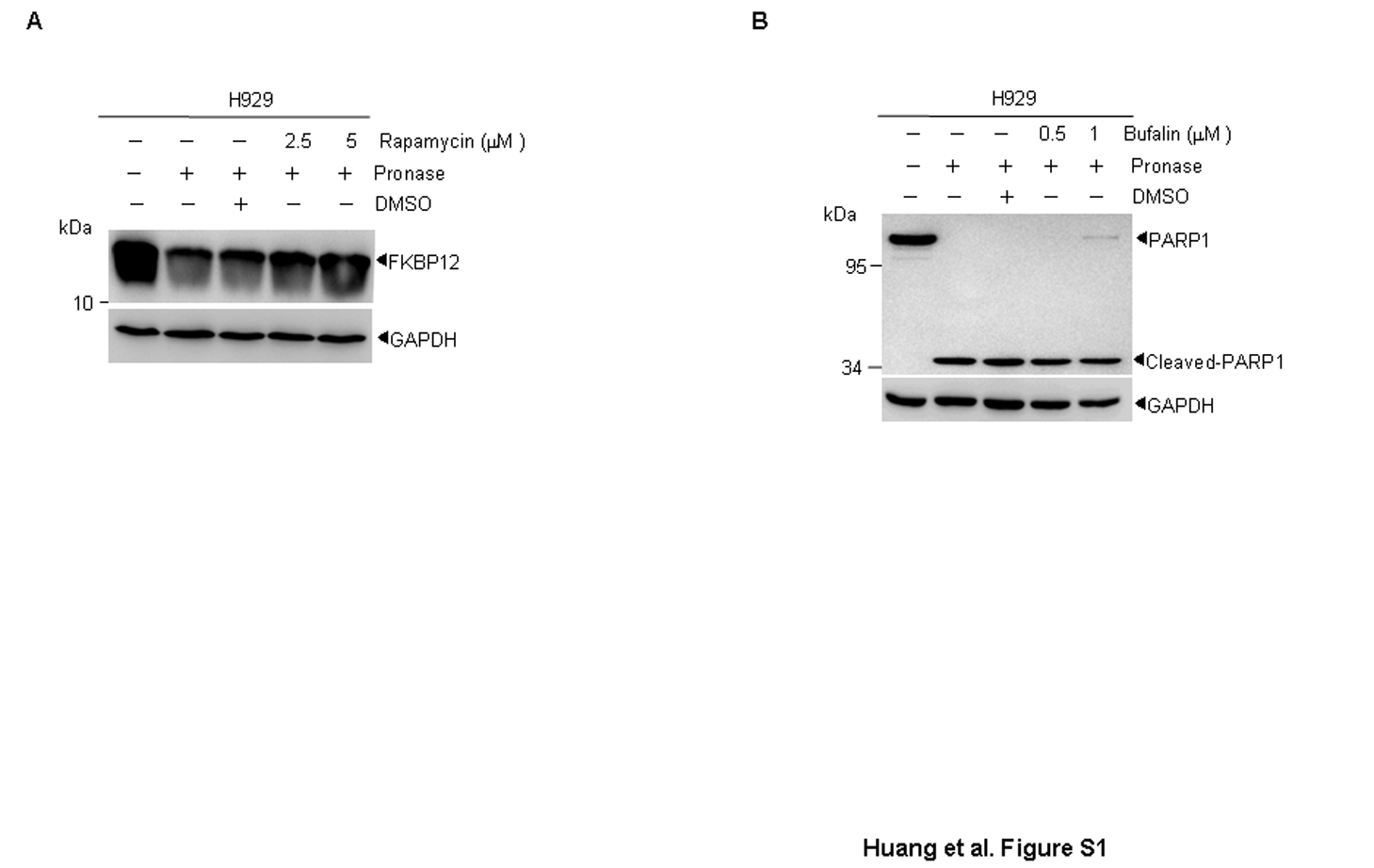

Supplement: Figure S1 — Using DARTS method to demonstrate the interaction between FKBP12 and rapamycin, or PARP1 and bufalin. H929 cell lysates were incubated with rapamycin (A) or bufalin (B) followed by digestion with pronase according to “Materials and Methods”. Then, the degree of FKBP12 and PARP1 protein degradation was determined by western blot. All experiments were repeated for three times. (TIF) [file pone.0066130.s001.tif]
